# Occurrence of multiple congenital anomalies in *Potamotrygon amandae* (Elasmobranchii, Potamotrygoninae) embryos, including the first report of bicephaly

**DOI:** 10.1111/jfb.70394

**Published:** 2026-03-18

**Authors:** Douglas de Castro Ribeiro, Raquel Lopes Queiroz, Cristiéle da Silva Ribeiro

**Affiliations:** ^1^ UNESP, Universidade Estadual Paulista Departamento de Biologia e Zootecnia, Laboratório de Paleontologia e Evolução de Ilha Solteira Ilha Solteira Brazil; ^2^ UNESP, Universidade Estadual Paulista Departamento de Biologia e Zootecnia, Laboratório de Estudos em Fisiologia Animal Ilha Solteira Brazil

**Keywords:** capture‐induced stress, Chondrichthyes, freshwater stingrays, Potamotrygonidae, teratology, upper Paraná River basin

## Abstract

Reports of teratogenic embryos in elasmobranchs have been documented in multiple species, with proposed aetiologies including environmental disturbances, genetic mutations, predation, exposure to endocrine‐disrupting compounds and maternal stress. Nevertheless, complex congenital malformations such as bicephaly remain exceptionally rare, particularly among freshwater stingrays of the family Potamotrygonidae. The present study constitutes the first record of bicephaly in the genus *Potamotrygon*, documenting multiple congenital anomalies in two embryos from a single litter. One female embryo exhibited incomplete fusion of the anterior margin of the right pectoral fin, a malformation previously reported in batoid fishes. The second case involved a male parapagus bicephalic twin; internal examination demonstrated duplicated brains and hearts, as well as partially individualized gastrointestinal tracts, whereas the reproductive and urinary systems were shared. Skeletal assessment revealed discrete chondrocrania and precaudal vertebrae, with spinal fusion occurring at the transitional caudal region. The underlying causes of these anomalies remain unresolved. Two primary hypotheses are discussed: (1) genetic alterations, including misexpression of critical developmental regulators such as Sonic hedgehog (Shh), which has been implicated in cephalic duplication and fin malformations; and (2) maternal physiological stress, potentially linked to anthropogenic factors such as recurrent capture events, which may disrupt embryogenesis. This report broadens the spectrum of known teratological conditions in freshwater elasmobranchs and underscores the importance of further research into the mechanistic basis of congenital anomalies in this group.

## 1 INTRODUCTION

Teratogenic embryos have already been reported in several elasmobranch species (e.g., Bornatowski & Abilhoa, 2009; Cabanillas‐Torpoco et al., 2023; Carmo & Fávaro, 2015; Delpiani et al., 2011; Galván‐Magaña et al., 2011; Guida et al., 2013; Hevia‐Hormazábal et al., 2011; Kanagusuku et al., 2020; Lopes et al., 2021; Mancini et al., 2006; Mejía‐Falla et al., 2011; Ramírez‐Amaro et al., 2019; Ribeiro‐Prado et al., 2008; Rosa‐Molinar & Williams, 1983; Sans‐Coma et al., 2016; Wosnick et al., 2019). The literature suggests multiple possible causes for these findings, including environmental changes, predation, exposure to endocrine disruptors, maternal nutritional factors, viral or parasitic infections and intrauterine mechanical stress (Galván‐Magaña et al., 2011; Guida et al., 2013; Silva & Casas, 2020). Additionally, intrinsic physiological factors related to an abortive response caused by physical constraint may also be associated with teratogenic development in sharks, stingrays and chimaeras (Rangel et al., 2020). Moreover, as most records concern viviparous species, it is plausible that maternal‐foetal interaction constitutes a major factor underlying the occurrence of these anomalies.

*Potamotrygon amandae* Loboda & Carvalho, 2013 is a freshwater stingray widely distributed in the Paraná–Paraguay and Amazon systems, with natural occurrence in the lower and middle regions of the Paraná River basin. However, the construction of the Itaipu Dam about 40 years ago destroyed the natural faunal barrier, the Sete Quedas Falls, allowing this and other aquatic species to access the upper portion of the basin (Garrone‐Neto et al., 2007; Langeani et al., 2007; Ota et al., 2018). *P. amandae* exhibits a unique colouration, greyish‐brown with bicoloured ocelli on the dorsal side of the disc; the ventral side is predominantly greyish or dark brown, with a lighter post‐buccal region (Loboda & Carvalho, 2013). It presents histotrophic viviparity as its reproductive mode (sensu Lodé, 2012), with a seasonal reproductive cycle of annual frequency, including vitellogenesis, ovulation and copulation during the dry season, followed by pregnancy and births during the rainy season (Silva et al., 2024). The gestation period and timing of birth, as in other species of the same genus, may last from 3 to 4 months (Charvet‐Almeida et al., 2005; Gama, 2013; Garrone‐Neto et al., 2007).

Here, we report morphological abnormalities in *P. amandae* embryos, describing the first occurrence of parapagus bicephalic twins in Potamotrygonin stingrays.

## 2 METHODS

Pregnant *P. amandae* females were collected in the Upper Paraná River region, in the ‘UHE Engenheiro Souza Dias’ reservoir (20°23′–20°46′S, 51°40′–51°22′W), between 2016 and 2024. The specimens were captured using cast nets and transported in polyethylene boxes filled with water to the Laboratorio de Estudos em Fisiologia Animal (LEFISA), Ilha Solteira, São Paulo, Brazil, where they were measured and weighed, and the offspring were analysed. The *P. amandae* vouchers were deposited in the Ichthyology Collection of the Instituto de Biociências Letras e Ciências exatas da Universidade Estadual Paulista ‘Júlio de Mesquita Filho’, São José do Rio Preto, São Paulo, Brazil (DZSJRP 21426) and in the Coleção de Zoologia do Laboratório de Paleontologia e Evolução de Ilha Solteira of the Universidade Estadual Paulista ‘Júlio Mesquita Filho’, Ilha Solteira, São Paulo, Brazil (LAPEISA‐Zoo 0116).

All experimental procedures followed the Ethical Conduct policies and guidelines in force in Brazil. Collection authorization was provided by Instituto Brasileiro de Meio Ambiente e Recursos Naturais Renováveis – IBAMA and Instituto Chico Mendes de Conservação da Biodiversidade – ICMBio (SISBIO #50019‐1). The study was also approved by the Comitê de Uso Animal da Universidade Estadual Paulista – UNESP and the Conselho Nacional de Controle de Experimentação Animal – CONCEA (CEUA #15/2018), and registered in the Sistema Nacional de Gestão do Patrimônio Genético e do Conhecimento Tradicional Associado – SISGEN (A001CBE).

Specimens were fixed in 4% formaldehyde solution for up to 48 h and transferred to 70% ethanol prior to dissection. Skeletal analysis was performed by double staining for cartilage and calcified elements/components using the protocols of Dingerkus and Uhler (1977) and Springer and Johnson (2000) and photographed using a Leica DM 2500 microscope equipped with a Leica DMC 2900 camera. Skeletal nomenclature is based on Carvalho et al. (2004). For the lateral line canal system, we follow the terminology of Maruska and Tricas (1998). The anatomical nomenclature (non‐skeletal) follows the Committee on Veterinary Gross Anatomical Nomenclature (Gasse, 2017).

## 3 RESULTS

In total, 75 pregnant females were examined, with a mean disc length of 41.9 ± 5.22 cm, a mean disc width of 37.4 ± 4.71 cm and a mean body mass of 3.05 ± 1.05 kg. All females were aborted after collection, with one to seven pups observed (mode = four). However, foetal anomalies were recorded in only one female. In this case, a female with 50.0 cm in disc length, 45.0 in disc width and a mass of 5.79 kg aborted seven partially developed offspring, with two specimens showing developmental anomalies.

**FIGURE 1:**
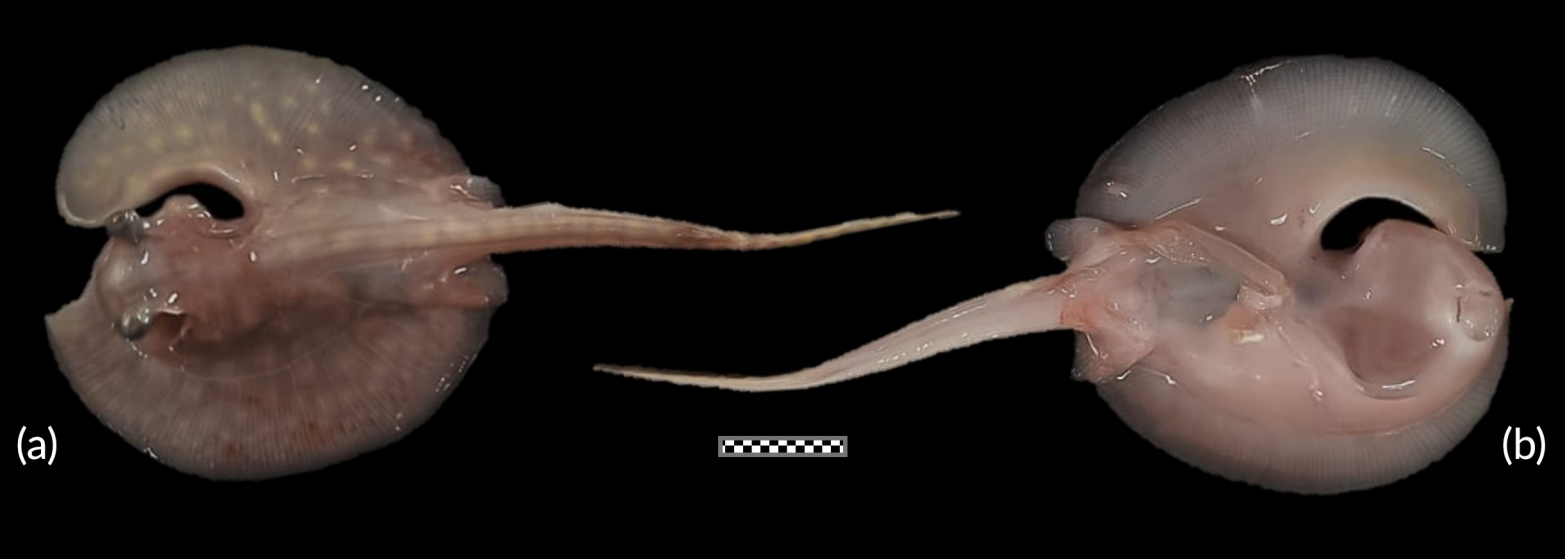
*Potamotrygon amandae* abnormal specimens (LAPEISA‐Zoo 00116). Female embryo with open disc in dorsal view (a) and ventral view (b). Scale bars = 5 mm.

A female embryo (Figure 1a,b) presented a malformation limited to the anterior part of the right side of the disc. These anomalies consisted of an anterior proximal margin of the pectoral fin not fused to the lateral portion of the head, from the tip of the snout to the anterior portion of the pectoral girdle; an underdeveloped spiracle; a caudal portion of the gill chamber deformed and poorly developed; and posterior gill arches that were poorly developed and potentially non‐functional. The rest of the body was apparently morphofunctional (Figure 1a,b). Notably, both abnormal embryos were larger than the normal embryos from the same litter (Table 1).

**TABLE 1:** Morphometric of abnormal (LAPEISA‐Zoo 00116) and mean of normal embryos of *Potamotrygon amandae*.

	Normal embryos	Abnormal embryos		
	(mean, SD)	Left twin	Right twin	Open disc
Mass	40.9–10.20	61.35		48.26
Total length	79.2–7.53	108.64	–	156.61
Disc length	39.3–4.02	52.63	–	76.91
Disc width	38.7–3.86	49.03	–	72.95
Tail length	40.60–14.49	58.06	–	82.85

*Note:* Mass values include yolk sac weight.

**FIGURE 2:**
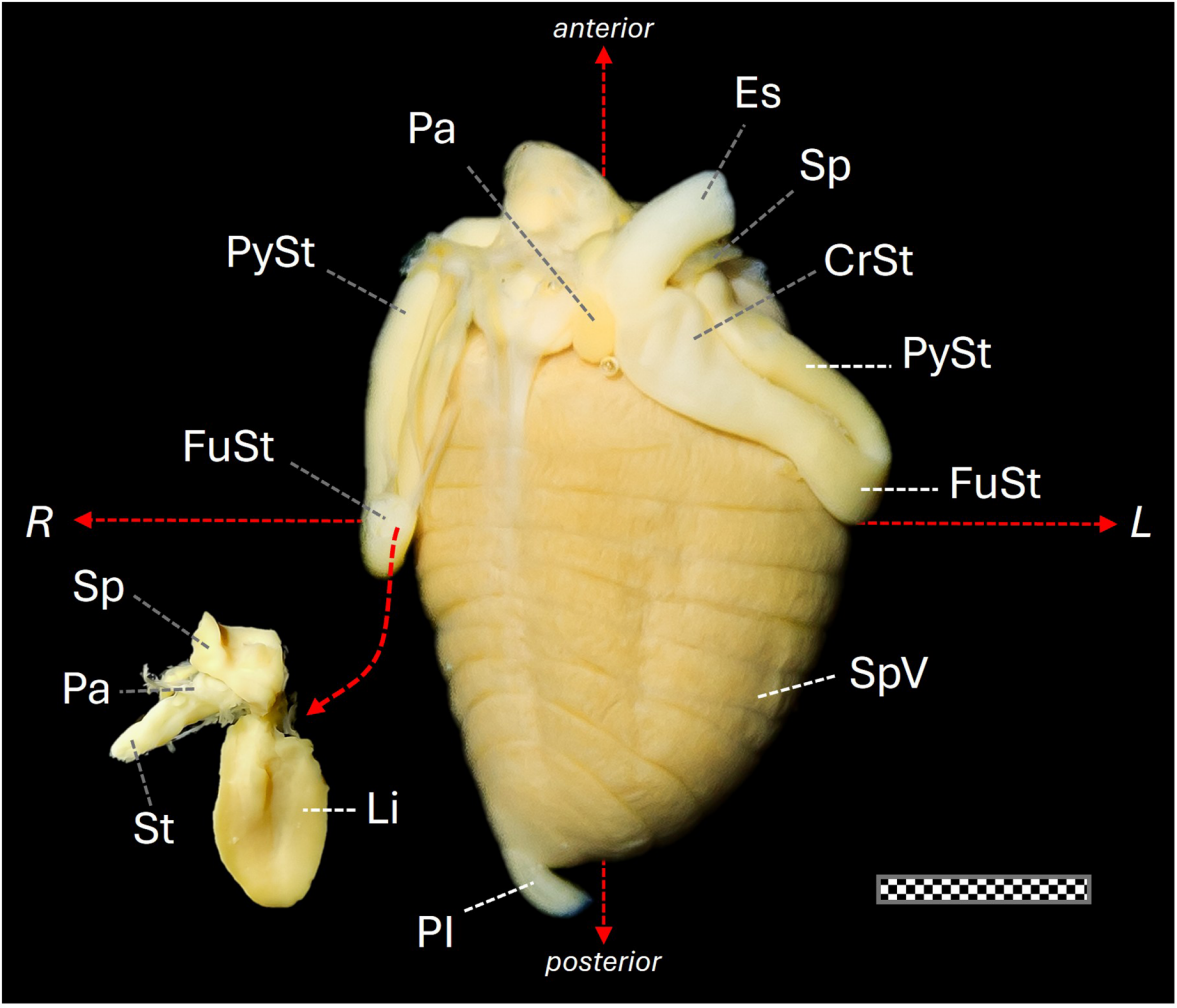
Spiral valve of *Potamotrygon amandae* conjugated bicephalic specimen (LAPEISA‐Zoo 00116), ventral view. CrSt, cranial stomach; Es, oesophagus; FuSt, fundic stomach; Li, liver*; Pa, pancreas*; PI, posterior intestine; PySt, pyloric stomach; Sp, spleen*; SpV, spiral valve; St, stomach*. Scale bars = 5 mm; * shown enlarged to highlight morphological details.

**FIGURE 3:**
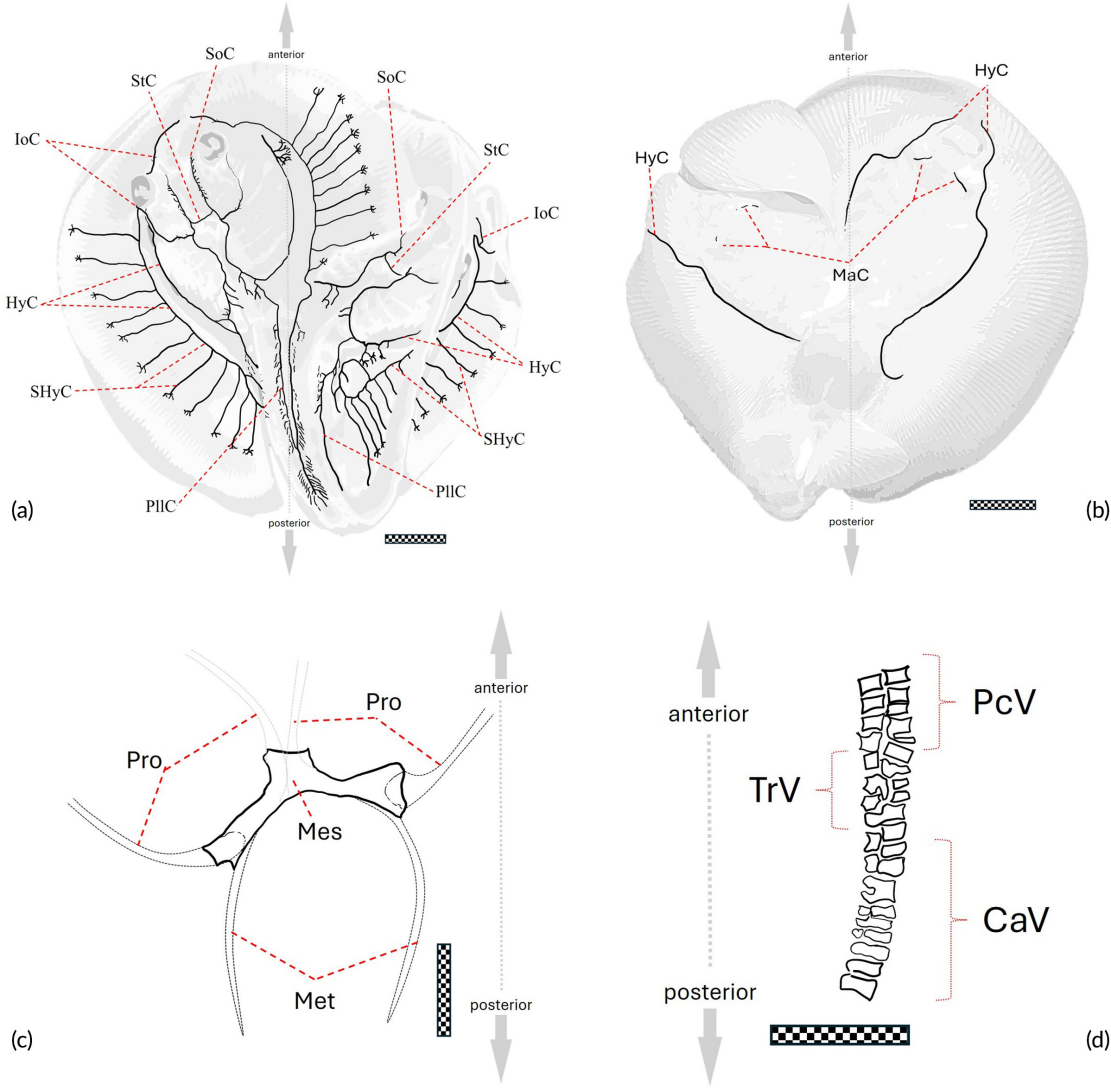
Schematic representation of the lateral line system and skeletal elements of the *Potamotrygon amandae* bicephalic specimen. (a) Dorsal view of the lateral line canals. (b) Ventral view of the lateral line canals. (c) Ventral view of the pectoral girdle elements (*Propterygium*, *Mesopterygium*, *Metapterygium*). (d) Ventral view of the vertebral column, shown enlarged to highlight morphological details, indicating precaudal (PcV), transitional (TrV) and caudal (CaV) vertebrae. CaV, caudal vertebrae; HyC, hyomandibular canal; IoC, infraorbital canal; MaC, mandibular canal; Mes, mesopterygium; Met, metapterygium; PcV, precaudal vertebrae; PllC, posterior lateral line canal; Pro, propterygium; SHyC, submandibular component of the hyomandibular canal, comprising the hyomandibular tubules that terminate in skin pores and the main hyomandibular canal branch that extends toward the posterior portion of the disc; SoC, supraorbital canal; StC, supratemporal canal; TrV, transitional vertebrae. Scale bars = 5 mm.

The second abnormal individual was observed in a male bicephalic specimen resulting from a twin pregnancy (Figure 1). An underdeveloped embryo was fused on the right side of the lateral pectoral girdle to its more developed twin (parapagus). Internally, both embryos had separate and morphofunctional olfactory tracts, brains, hearts, eyes, mouths (lips, oral papillae and rostral labium), branchial chambers and spiracles. Most of the gastrointestinal tract, reproductive tract and urinary tract were located in the left twin. The gastrointestinal tract was partially separated, with the oesophagus, stomach, pylorus, duodenum, spleen, pancreas and liver (right side underdeveloped) individualized (Figure 2). The digestive tract contained two independent stomachs, one on each side (Figure 2). The reproductive and urinary tracts were shared and apparently morphofunctional. The lateral line system was well developed in the left twin; however, the ventral components, including the supraorbital canal, posterior abdominal portion of the hyomandibular canal, posterior caudal canal and mandibular canal, were poorly developed in the right twin (Figure 3a,b).

Skeletal analysis revealed that the chondrocranium, splanchnocranium and precaudal vertebrae were separate and had normal morphology. The fusion between twins occurred through the lateral surfaces of the mesopterygium, in the midline of the abdomen, with two pairs of propterygia and only one pair of metapterygia observed, all well developed (Figure 3c). Spinal fusion occurred in the transitional region between precaudal and caudal vertebrae, with normal vertebral centres after caudal vertebrae V6 and V8 (Figure 3d). Morphometric data of the embryos are available in Table 1.

## 4 DISCUSSION

The non‐fusion of the pectoral fins to the head is one of the most frequently documented disc anomalies in batoids, particularly in stingrays (Blanco‐Parra & Ñino‐Torres, 2011; Mejía‐Falla et al., 2011; Schmid et al., 2019). Similar unfused‐disc malformations have been reported in freshwater species such as *Potamotrygon marquesi* and *Potamotrygon motoro* (Rosa et al., 1996; Silva & Casas, 2020), indicating that this condition is not restricted to a particular habitat type. Although disc non‐fusion may impair swimming and feeding, some juveniles of other batoids, such as *Myliobatis chilensis*, have been documented surviving with reduced or altered pectoral fin attachment, without evident mobility loss (Céspedes et al., 2023). Morphological compensations associated with fin malformations, including spiracle repositioning or gill rearrangement, have been described in *Trygonorrhina dumerilii* (Guida et al., 2013) and *Prionace glauca* (Galván‐Magaña et al., 2011). In *Zapteryx brevirostris*, severe teratogenic effects such as the absence of gill structures and impaired yolk absorption reinforce the susceptibility of batoids to cephalic and axial developmental disorders (Wosnick et al., 2019). Considering these reports, the malformation observed in *P. amandae* aligns with patterns documented across multiple elasmobranch lineages. These studies suggest that such defects may arise from environmental stressors, nutritional imbalance, toxic exposure, parasitism or intrinsic embryonic developmental instability, indicating that more than one causative mechanism could underlie the anomaly observed here.

As far as is currently known, there have been no records of bicephaly in species of the genus *Potamotrygon*. Although other congenital malformations have already been reported in stingrays of this group, including alterations in fins, such as absence or detachment from the cephalic region, claspers and other morphological structures (e.g., Rosa et al., 1996; Silva & Casas, 2020), this is the first report of bicephalism in Potamotrygoninae stingrays. The available records of bicephaly in elasmobranchs have been concentrated in sharks, such as *Galeus atlanticus*, *Galeorhinus galeus*, *Carcharhinus porosus*, *P. glauca*, *Carcharhinus leucas* and *Squalus acanthias* (Delpiani et al., 2011; Goto et al., 1981; Lopes et al., 2021; Muñoz‐Osorio et al., 2013; Sans‐Coma et al., 2016; Wagner et al., 2013), with only one description in stingrays, for the marine species *T. dumerilii* (Guida et al., 2013), showing that the condition, although rare, may occur in different lineages.

Cases of bicephaly in elasmobranchs have generally been restricted to external descriptions of duplicated heads or general cephalic deformities, with duplication of major organs (e.g., the brain and heart) still scarcely documented (Rosa‐Molinar & Williams, 1983; Sans‐Coma et al., 2016). The thorough analysis and detailed description of the asymmetric development of the lateral line system and the individualization of multiple components of the gastrointestinal tract in this bicephalic specimen appear to deepen existing knowledge and may represent an advance in the anatomical characterization of such anomalies in elasmobranchs.

The observation of partial vertebral column fusion reported here contrasts with findings in other chondrichthyan species. In these, duplication of the vertebral column has most often been described in cases of bicephaly. For example, in a bicephalic *T. dumerilii* found in Australian waters, magnetic resonance imaging (MRI) revealed two separate vertebral columns running parallel along the entire body and tail, diverging into two well‐formed heads (Guida et al., 2013). Similarly, in a bicephalic embryo of *G. atlanticus*, the authors observed two parallel notochords and two dorsal vertebral columns extending throughout the body (Sans‐Coma et al., 2016). Additionally, a bicephalic specimen of *G. galeus* presented two separate vertebral columns, with radiographs indicating differences in the number of vertebrae between the right and left individuals. These observations suggest that, rather than partial fusion as found in the present study, duplication of axial structures occurs, resulting in two parallel vertebral columns supporting the two heads in the bicephalic individual (Guida et al., 2013; Pastén‐Marambio et al., 2018; Rosa‐Molinar & Williams, 1983).

Although the exact causes of the anomalies found remain uncertain, two main hypotheses have been suggested: (1) genetic anomalies, since the separation of the pectoral fins from the head is considered a natural developmental mutation (Guida et al., 2013; Lima et al., 2024; Mejía‐Falla et al., 2011; Pastén‐Marambio et al., 2018; Sans‐Coma et al., 2016), given that most embryos in the same uterus were normal and the capture areas were preserved. Additionally, the same condition may be linked to anomalies in the expression of key embryological genes, including *Sox9, Grem1, Fgf8, Hand2* and, especially, *Sonic hedgehog (Shh)* (Lima et al., 2024; Pastén‐Marambio et al., 2018; Rosa‐Molinar & Williams, 1983), which have also been cited as potential causes of developmental anomalies in elasmobranchs, such as bicephaly; (2) physiological stress induced by natural and anthropogenic actions, increasing the levels of stress hormones such as 1‐α‐hydroxycorticosterone in elasmobranchs (Armour et al., 1993), thereby disrupting embryogenesis during the early stages of gestation; repeated capture events (e.g., fisheries interactions) may further exacerbate this effect (Guida et al., 2013; Rangel et al., 2020; Silva & Casas, 2020). Similarly, the size asymmetry observed between the twins may indicate impaired maternal–foetal nutrient transfer, a process known to exhibit high susceptibility to physiological and environmental perturbations in elasmobranch embryos (Lyons & Wynne‐Edwards, 2021). Moreover, hydroelectric dam construction can contribute to developmental disruption by reshaping habitat structure, altering hydrological and physicochemical conditions and increasing exposure to intrinsic contaminants released or mobilized through hydroelectric dam operation (e.g., polychlorinated biphenyls, chlorinated hydrocarbons, heavy metals), factors that have been associated with elevated frequencies of developmental anomalies in aquatic taxa (Ferraz et al., 2021). Regardless of the cause, these malformations may seriously compromise the mobility and survival of affected individuals in the wild, limiting their ability to hunt, reproduce and escape predators.

Despite the apparently functional morphology of the collected embryos, bicephalic or dicephalic chondrichthyans consistently show that such individuals are either stillborn or die shortly after birth, with malformations of the head and axial skeleton that severely compromise survival (Guida et al., 2013; Muñoz‐Osorio et al., 2013; Wagner et al., 2013). On the contrary, individuals presenting only an open disc have greater chances of development; however, there are no reports of individuals with this condition (‘bat rays’ in the aquarium hobby) in natural environments, only in closed captive systems (Oldfield, 2007). The absence of reports of complex anomalies in *Potamotrygon* suggests that this condition is particularly unknown in freshwater stingrays, making the present finding relevant as the first description of this condition in the group and expanding the knowledge on teratological variability in freshwater elasmobranchs.

## AUTHOR CONTRIBUTIONS

Douglas de Castro Ribeiro contributed to conceptualization, methodology, funding acquisition, writing original draft and writing review and editing. Raquel Lopes Queiroz contributed to resources, writing original draft and writing review and editing. Cristiéle da Silva Ribeiro contributed to conceptualization, methodology, funding acquisition, writing original draft and writing review and editing.

## FUNDING INFORMATION

Coordenação de Aperfeiçoamento de Pessoal de Nível Superior – Brasil (CAPES) − Finance Code 001, Master scholarship and Research Grant (PROAP).

## CONFLICT OF INTEREST STATEMENT

The authors declare no potential conflict of interests.

## Data Availability

The data that support the findings of this study are available from the corresponding author upon reasonable request.
